# *N*,*N*′-Di­hydroxy­benzene-1,2:4,5-tetra­carboximide dihydrate

**DOI:** 10.1107/S1600536813016991

**Published:** 2013-06-26

**Authors:** Roberto Centore, Antonio Carella

**Affiliations:** a Dipartimento di Scienze Chimiche, Università degli Studi di Napoli ’Federico II’, Complesso di Monte S. Angelo, Via Cinthia, 80126 Napoli, Italy

## Abstract

In the title compound, C_10_H_4_N_2_O_6_·2H_2_O, the organic mol­ecule has crystallographically imposed inversion symmetry. The atoms of the three fused rings of the mol­ecule are coplanar within 0.0246 (8) Å, while the two hy­droxy O atoms are displaced from the mean plane of the mol­ecule by 0.127 (1) Å. In the crystal, infinite near-planar layers of close-packed mol­ecules are formed by hydrogen bonding between water O—H donor groups and carbonyl O-atom acceptors, and by weak inter­actions between C—H donor groups and water O-atom acceptors. The layers are parallel to the {102} family of planes. The stacked planes are held together by hydrogen bonding between N—OH donor groups and water O-atom acceptors.

## Related literature

For semiconductor, optoelectronic and piezoelectric materials containing heterocycles, see: Centore, Ricciotti *et al.* (2012 ▶); Centore, Concilio *et al.* (2012 ▶). For the structural analysis of conjugation in organic mol­ecules containing heterocycles, see: Carella *et al.* (2004 ▶). For the crystal packing of heterocycles containing nitro­gen, see: Centore *et al.* (2013*a* ▶,*b* ▶). For the crystal engineering of structures containing stacked infinite planar layers, see: Centore, Causà *et al.* (2013 ▶). For the principle of close packing in organic crystallography, see: Kitaigorodskii (1961 ▶).
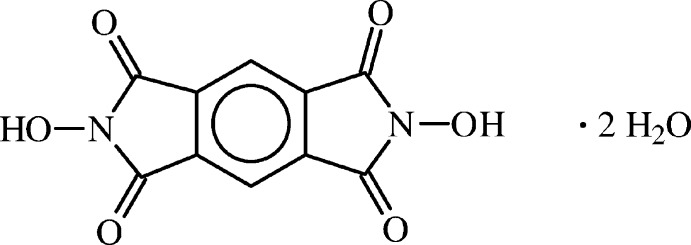


## Experimental

### 

#### Crystal data

C_10_H_4_N_2_O_6_·2H_2_O
*M* *_r_* = 284.18
Monoclinic, 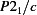

*a* = 6.874 (3) Å
*b* = 10.189 (5) Å
*c* = 8.099 (4) Å
β = 106.58 (2)°
*V* = 543.7 (4) Å^3^
*Z* = 2
Mo *K*α radiation
μ = 0.15 mm^−1^
*T* = 293 K
0.40 × 0.40 × 0.30 mm

#### Data collection

Bruker–Nonius KappaCCD diffractometer
Absorption correction: multi-scan (*SADABS*; Bruker, 2001 ▶) *T*_min_ = 0.941, *T*_max_ = 0.955
4484 measured reflections
1231 independent reflections
1100 reflections with *I* > 2σ(*I*)
*R*_int_ = 0.035

#### Refinement

*R*[*F* ^2^ > 2σ(*F* ^2^)] = 0.036
*wR*(*F* ^2^) = 0.100
*S* = 1.09
1231 reflections
100 parameters
H atoms treated by a mixture of independent and constrained refinement
Δρ_max_ = 0.22 e Å^−3^
Δρ_min_ = −0.25 e Å^−3^

### 

Data collection: *COLLECT* (Nonius, 1999 ▶); cell refinement: *DIRAX/LSQ* (Duisenberg *et al.*, 2000 ▶); data reduction: *EVALCCD* (Duisenberg *et al.*, 2003 ▶); program(s) used to solve structure: *SIR97* (Altomare *et al.*, 1999 ▶); program(s) used to refine structure: *SHELXL97* (Sheldrick, 2008 ▶); molecular graphics: *ORTEP-3 for Windows* (Farrugia, 2012 ▶) and *Mercury* (Macrae *et al.*, 2006 ▶); software used to prepare material for publication: *WinGX* (Farrugia, 2012 ▶).

## Supplementary Material

Crystal structure: contains datablock(s) global, I. DOI: 10.1107/S1600536813016991/rz5073sup1.cif

Structure factors: contains datablock(s) I. DOI: 10.1107/S1600536813016991/rz5073Isup2.hkl

Supplementary material file. DOI: 10.1107/S1600536813016991/rz5073Isup3.cml

Additional supplementary materials:  crystallographic information; 3D view; checkCIF report

## Figures and Tables

**Table 1:** Hydrogen-bond geometry (Å, °)

*D*—H⋯*A*	*D*—H	H⋯*A*	*D*⋯*A*	*D*—H⋯*A*
O3—H3⋯O4^i^	0.886 (19)	1.768 (19)	2.6516 (18)	175.2 (17)
O4—H4*A*⋯O2^ii^	0.896 (19)	2.15 (2)	3.0441 (17)	172.4 (16)
O4—H4*B*⋯O1^iii^	0.909 (19)	1.990 (19)	2.8879 (16)	169.3 (15)
C1—H1⋯O4	0.93	2.40	3.280 (2)	158

Symmetry codes: (i) 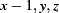; (ii) 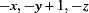; (iii) 
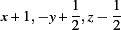
.

**Fig. 1.**
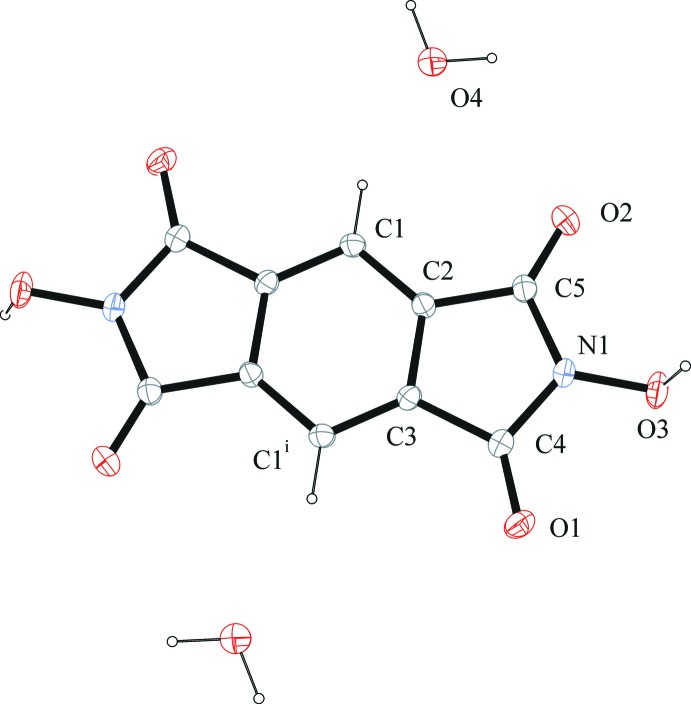
ORTEP view of the molecular structure of the title compound. Displacement ellipsoids are drawn at the 30% probability level. Symmetry code: (i) = -x, -y, -z.

**Fig. 2.**
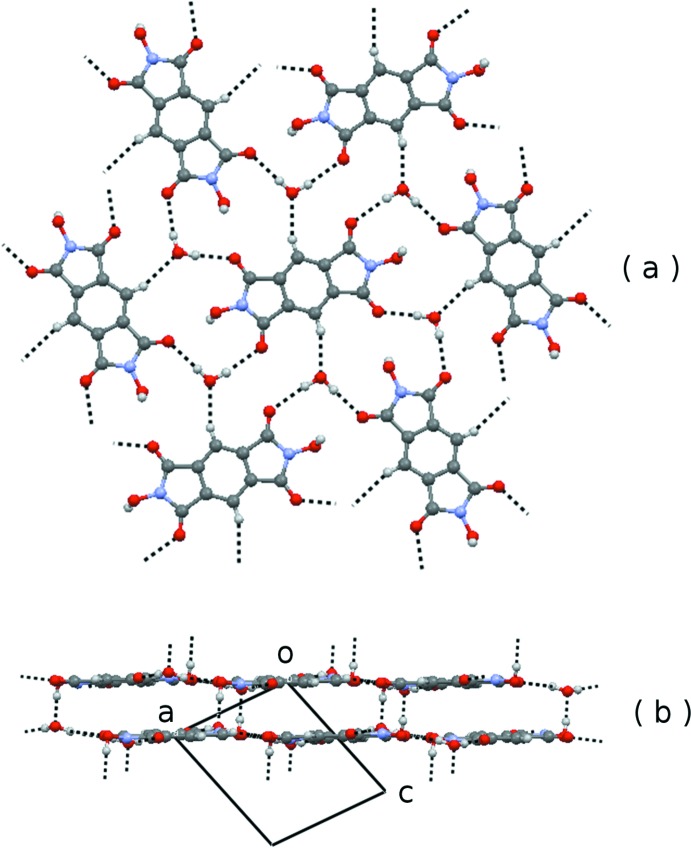
Partial crystal packing of the title compound, showing some H bonding patterns. H bonds are represented by dashed lines; (a) front view of a layer of H bonded molecules; (b) edge view, along b, of two adjacent layers.

## supplementary crystallographic information

### Comment

The crystal engineering of structures containing stacked infinite planar layers of H bonded aromatic molecules is a hot research topic because of the potential interest of such structures as advanced materials in organic electronics, optoelectronics and photonics (Centore, Causà *et al.* 2013). Conjugated heterocyclic aromatic compounds are often used as building blocks for assembling active molecules for those advanced applications (Carella *et al.*, 2004; Centore, Concilio *et al.*, 2012). Aromatic diimides, in particular, are well known for their outstanding properties as *n*-type organic semiconductors (Centore, Ricciotti *et al.*, 2012). Following these issues and our basic interest for crystal structures of conjugated heteroaromatic compounds conditioned by the formation of strong and weak H bonds (Centore *et al.*, 2013*a*; 2013*b*) we report the structural investigation of the title compound, which is the dihydrate form of the *N,N'*-dihydroxy derivative of the simplest aromatic bis(imide).

The molecular structure is shown in Fig. 1. The organic molecule lies in special position on inversion centres and has point symmetry *C_i_*. The atoms of the three fused rings of the molecule are coplanar within 0.0246 (8) Å, while the two hydroxy oxygen atoms are out of the average plane of the molecule by ±0.127 (1) Å. This is related with the torsion angle C2–C5–N1–O3 which is 171.9 (1)°, and would suggest a slight degree of pyramidalization at N1 (the sum of the valence angles around N1 is 359.1 (2)°). The two hydroxy H atoms are off the molecular plane because of the torsion angle C5–N1–O3–H3 = 80 (1)°.

Each organic molecule is in-plane surrounded by six water molecules. With four of them there are H bonds between O–H donors of the water molecules and carbonyl O acceptors of the diimide molecule. With the remaining two water molecules, there are weak interactions involving C–H donor and water O acceptor. Infinite planar layers of close-packed H bonded molecules are formed in this way, Fig. 2(*a*), in which ring patterns *R*^4^_4_(14) and *R*^4^_4_(15) are easily spotted. The layers are parallel to the family of planes with Miller indices (1 0 2), Fig. 2(*b*), and, in fact, the reflection (1 0 2) is the most intense of the whole diffraction pattern because, with the exception for the N–OH hydrogen atoms, all the atoms of the two molecules lie onto those planes. The stacked layers are held together by H bonds involving N–OH donors of a layer and water O acceptors of adjacent layers and this may account for the short interplanar spacing, *d*_102_ = 2.99 Å. As it is clear from Fig. 2(*b*), adjacent stacked layers are related by a 2_1_ screw operation, which is a very efficient way to fulfill the Kitaigorodskii's "bumps in hollows" golden rule of the close packing of layers (Kitaigorodskii, 1961).

### Experimental

Hydroxylamine hydrochloride (3.18 g, 45.8 mmol) was added to pyridine (25 ml) and stirred at room temperature for 10 min until a clear solution was obtained. Pyromellitic anhydride (5.00 g, 22.9 mmol) was added and the solution was refluxed overnight. The solution was cooled to room temperature and the solid precipitate was filtered and washed with methanol and dried in oven at 120 °C for three days. Then it was poured in 100 ml of a solution methanol/conc. HCl (9:1 *v*/*v*). The suspension was left on stirring at gentle boiling for 1 h and then cooled to room temperature. The solid precipitate was filtered. The yield was 1.70 g (30%). ^1^H-NMR (DMSO-d^6^, 200 MHz): 8.11 (2*H*, s, C–H); 11.14 (2*H*, s, N–OH).

Single crystals for X-ray analysis were obtained by slow evaporation of an ethanol/water (1:1 *v*/*v*) solution.

### Refinement

The H atoms bonded to O atoms were located in a difference Fourier map and their coordinates were refined. The H atom on benzene ring was generated stereochemically and was refined by the riding model. For all H atoms *U*_iso_(H) = 1.2*U*_eq_(C, O) was assumed.

### Crystal data

**Table:**

C_10_H_4_N_2_O_6_·2H_2_O	*F*(000) = 292
*M**_r_* = 284.18	*D*_x_ = 1.736 Mg m^−^^3^
Monoclinic, *P*2_1_/*c*	Mo *K*α radiation, λ = 0.71073 Å
Hall symbol: -P 2ybc	Cell parameters from 150 reflections
*a* = 6.874 (3) Å	θ = 4.9–23.6°
*b* = 10.189 (5) Å	µ = 0.15 mm^−^^1^
*c* = 8.099 (4) Å	*T* = 293 K
β = 106.58 (2)°	Prism, yellow
*V* = 543.7 (4) Å^3^	0.40 × 0.40 × 0.30 mm
*Z* = 2	

### Data collection

**Table:**

Bruker–Nonius KappaCCD diffractometer	1231 independent reflections
Radiation source: normal-focus sealed tube	1100 reflections with *I* > 2σ(*I*)
Graphite monochromator	*R*_int_ = 0.035
Detector resolution: 9 pixels mm^-1^	θ_max_ = 27.5°, θ_min_ = 3.1°
CCD rotation images, thick slices scans	*h* = −8→8
Absorption correction: multi-scan (*SADABS*; Bruker, 2001)	*k* = −11→13
*T*_min_ = 0.941, *T*_max_ = 0.955	*l* = −10→9
4484 measured reflections	

### Refinement

**Table:**

Refinement on *F*^2^	Primary atom site location: structure-invariant direct methods
Least-squares matrix: full	Secondary atom site location: difference Fourier map
*R*[*F*^2^ > 2σ(*F*^2^)] = 0.036	Hydrogen site location: inferred from neighbouring sites
*wR*(*F*^2^) = 0.100	H atoms treated by a mixture of independent and constrained refinement
*S* = 1.09	*w* = 1/[σ^2^(*F*_o_^2^) + (0.0519*P*)^2^ + 0.137*P*] where *P* = (*F*_o_^2^ + 2*F*_c_^2^)/3
1231 reflections	(Δ/σ)_max_ < 0.001
100 parameters	Δρ_max_ = 0.22 e Å^−^^3^
0 restraints	Δρ_min_ = −0.25 e Å^−^^3^

### Special details

**Table:**

Geometry. All e.s.d.'s (except the e.s.d. in the dihedral angle between two l.s. planes) are estimated using the full covariance matrix. The cell e.s.d.'s are taken into account individually in the estimation of e.s.d.'s in distances, angles and torsion angles; correlations between e.s.d.'s in cell parameters are only used when they are defined by crystal symmetry. An approximate (isotropic) treatment of cell e.s.d.'s is used for estimating e.s.d.'s involving l.s. planes.
Refinement. Refinement of *F*^2^ against ALL reflections. The weighted *R*-factor *wR* and goodness of fit *S* are based on *F*^2^, conventional *R*-factors *R* are based on *F*, with *F* set to zero for negative *F*^2^. The threshold expression of *F*^2^ > σ(*F*^2^) is used only for calculating *R*-factors(gt) *etc*. and is not relevant to the choice of reflections for refinement. *R*-factors based on *F*^2^ are statistically about twice as large as those based on *F*, and *R*- factors based on ALL data will be even larger.

### Fractional atomic coordinates and isotropic or equivalent isotropic displacement parameters (Å^2^)

**Table:**

	*x*	*y*	*z*	*U*_iso_*/*U*_eq_	
C1	0.10245 (17)	0.10697 (12)	−0.05619 (15)	0.0244 (3)	
H1	0.1688	0.1760	−0.0924	0.029*	
C2	−0.05331 (17)	0.12613 (11)	0.01756 (15)	0.0230 (3)	
C3	−0.15149 (15)	0.02254 (11)	0.07171 (14)	0.0230 (3)	
C4	−0.31036 (17)	0.07755 (12)	0.14333 (15)	0.0254 (3)	
C5	−0.14618 (17)	0.25083 (12)	0.05285 (15)	0.0258 (3)	
N1	−0.29927 (15)	0.21147 (11)	0.12231 (14)	0.0287 (3)	
O1	−0.42607 (13)	0.02174 (10)	0.20623 (12)	0.0346 (3)	
O2	−0.10464 (15)	0.36247 (8)	0.02877 (13)	0.0360 (3)	
O3	−0.40708 (15)	0.29756 (10)	0.19205 (13)	0.0367 (3)	
H3	−0.499 (3)	0.3314 (17)	0.103 (2)	0.044*	
O4	0.32117 (15)	0.38853 (10)	−0.08642 (14)	0.0367 (3)	
H4A	0.249 (2)	0.4570 (19)	−0.067 (2)	0.044*	
H4B	0.387 (3)	0.4151 (18)	−0.163 (2)	0.044*	

### Atomic displacement parameters (Å^2^)

**Table:**

	*U*^11^	*U*^22^	*U*^33^	*U*^12^	*U*^13^	*U*^23^
C1	0.0234 (5)	0.0230 (6)	0.0280 (6)	−0.0016 (4)	0.0092 (4)	0.0017 (5)
C2	0.0226 (5)	0.0207 (5)	0.0255 (5)	0.0009 (4)	0.0063 (4)	0.0001 (4)
C3	0.0198 (5)	0.0255 (6)	0.0246 (5)	0.0000 (4)	0.0077 (4)	−0.0006 (4)
C4	0.0231 (5)	0.0285 (6)	0.0248 (5)	0.0012 (4)	0.0074 (4)	−0.0019 (4)
C5	0.0268 (6)	0.0245 (6)	0.0258 (5)	0.0030 (4)	0.0072 (4)	−0.0003 (5)
N1	0.0303 (5)	0.0264 (6)	0.0335 (5)	0.0068 (4)	0.0156 (4)	−0.0007 (4)
O1	0.0314 (5)	0.0382 (6)	0.0405 (5)	−0.0032 (4)	0.0202 (4)	−0.0014 (4)
O2	0.0436 (6)	0.0221 (5)	0.0451 (6)	0.0019 (4)	0.0170 (5)	0.0012 (4)
O3	0.0383 (5)	0.0379 (6)	0.0369 (5)	0.0155 (4)	0.0157 (4)	−0.0047 (4)
O4	0.0379 (5)	0.0324 (5)	0.0436 (6)	0.0011 (4)	0.0178 (4)	0.0028 (4)

### Geometric parameters (Å, º)

**Table:**

C1—C3^i^	1.3766 (17)	C4—N1	1.3799 (18)
C1—C2	1.3806 (17)	C5—O2	1.2022 (16)
C1—H1	0.9300	C5—N1	1.3866 (16)
C2—C3	1.3895 (16)	N1—O3	1.3699 (13)
C2—C5	1.4858 (16)	O3—H3	0.886 (19)
C3—C1^i^	1.3766 (17)	O4—H4A	0.896 (19)
C3—C4	1.4848 (16)	O4—H4B	0.909 (19)
C4—O1	1.2028 (15)		
			
C3^i^—C1—C2	114.54 (11)	O1—C4—C3	129.49 (12)
C3^i^—C1—H1	122.7	N1—C4—C3	104.62 (10)
C2—C1—H1	122.7	O2—C5—N1	125.62 (11)
C1—C2—C3	122.39 (11)	O2—C5—C2	130.01 (11)
C1—C2—C5	129.26 (11)	N1—C5—C2	104.37 (10)
C3—C2—C5	108.35 (11)	O3—N1—C4	121.80 (10)
C1^i^—C3—C2	123.07 (11)	O3—N1—C5	123.00 (11)
C1^i^—C3—C4	128.62 (11)	C4—N1—C5	114.27 (10)
C2—C3—C4	108.31 (11)	N1—O3—H3	105.0 (11)
O1—C4—N1	125.88 (11)	H4A—O4—H4B	107.4 (16)
			
C3^i^—C1—C2—C3	0.13 (18)	C3—C2—C5—O2	178.61 (12)
C3^i^—C1—C2—C5	−179.51 (11)	C1—C2—C5—N1	178.07 (11)
C1—C2—C3—C1^i^	−0.1 (2)	C3—C2—C5—N1	−1.61 (13)
C5—C2—C3—C1^i^	179.57 (10)	O1—C4—N1—O3	8.09 (19)
C1—C2—C3—C4	−179.69 (10)	C3—C4—N1—O3	−172.06 (10)
C5—C2—C3—C4	0.02 (12)	O1—C4—N1—C5	177.34 (11)
C1^i^—C3—C4—O1	1.9 (2)	C3—C4—N1—C5	−2.80 (13)
C2—C3—C4—O1	−178.56 (12)	O2—C5—N1—O3	−8.29 (19)
C1^i^—C3—C4—N1	−177.92 (11)	C2—C5—N1—O3	171.92 (10)
C2—C3—C4—N1	1.59 (12)	O2—C5—N1—C4	−177.40 (12)
C1—C2—C5—O2	−1.7 (2)	C2—C5—N1—C4	2.81 (13)

Symmetry code: (i) −*x*, −*y*, −*z*.

### Hydrogen-bond geometry (Å, º)

**Table:**

*D*—H···*A*	*D*—H	H···*A*	*D*···*A*	*D*—H···*A*
O3—H3···O4^ii^	0.886 (19)	1.768 (19)	2.6516 (18)	175.2 (17)
O4—H4*A*···O2^iii^	0.896 (19)	2.15 (2)	3.0441 (17)	172.4 (16)
O4—H4*B*···O1^iv^	0.909 (19)	1.990 (19)	2.8879 (16)	169.3 (15)
C1—H1···O4	0.93	2.40	3.280 (2)	158

Symmetry codes: (ii) *x*−1, *y*, *z*; (iii) −*x*, −*y*+1, −*z*; (iv) *x*+1, −*y*+1/2, *z*−1/2.
